# Supplementary material for: Swelling-Resistant, Crosslinked Polyvinyl Alcohol Membranes with High ZIF-8 Nanofiller Loadings as Effective Solid Electrolytes for Alkaline Fuel Cells
Source: Nanomaterials (Basel). 2022 Mar 4;12(5):865. doi: 10.3390/nano12050865 (PMC8912677; doi:10.3390/nano12050865)
Supplement: Supplementary file 1 [file nanomaterials-12-00865-s001.zip › nanomaterials-1566896-supplementary.pdf]

nanomaterials

MDPI

Supplementary information

Swelling-Resistant, Crosslinked Polyvinyl Alcohol Membranes with High ZIF-8 Nanofiller Loadings as Effective Solid Electrolytes for Alkaline Fuel Cells

Po-Ya Hsu [1,†], Ting-Yu Hu [1,†], Selvaraj Rajesh Kumar [1], Kevin C.-W. Wu [2,3*] and Shingjiang Jessie Lue [1,4,5,*]

1 Department of Chemical and Materials Engineering, Chang Gung University, Guishan District, Taoyuan City 333, Taiwan; mini50636@gmail.com (P.-Y.H.); hu70308@gmail.com (T.-Y.H.); rajeshkumarnst@gmail.com (S.R.K.)
2 Department of Chemical Engineering, National Taiwan University, Taipei City 106, Taiwan
3 Institute of Biomedical Engineering and Nanomedicine, National Health Research Institutes, Miaoli County 350, Taiwan
4 Department of Safety, Health and Environment Engineering, Ming Chi University of Technology, Taishan, New Taipei City 243, Taiwan
5 Division of Joint Reconstruction, Department of Orthopedics, Chang Gung Memorial Hospital, Linkou, Taoyuan City 333, Taiwan
* Correspondence: kevinwu@ntu.edu.tw (K.C.-W.W.); jessie@mail.cgu.edu.tw (S.J.L.); Tel.: +886-3366-3064 (K.C.-W.W.); +866-3211-8800 (ext. 5489) (S.J.L.); Fax: +886-3211-8700 (S.J.L.)
† Equivalent contribution to first author.

Table S1. Comparison of acid and alkaline-based PVA composite electrolytes for direct methanol fuel cells. .

Electrolyte types	Membranes	Filler loading (wt%)	MeOH Con. (M)	Catalyst load (mg cm^{-2})		Temperature (°C)	Peak power density (Pmax) mW cm^{-2}	Source
				Anode (Pt-Ru)	Cathode (Pt)			
Acid-doped	CBA/Nafion-PVA	-	2	4	4	80	68.7	Li et al. [1] (2018)
	PVA/alumina	20	1	2	2	60	6.3	Imaan et al. [2] (2021)
	PVA/SSA-GO	1	1	3	1	50	19.5	Castell et al. [3] (2020)
	PVA coated Nafion	-	7	3.5	3.5	25	30	Rao et al. [4] (2021)
Without KOH	PVA/AA-TiO_2/GA	3	2	2	2	70	58.3	Mohanapriya et al. [5] (2016)
	CBC/PVA/zeolite	10	0.74	30wt%[a]	20wt%[a]	80	6.5	Altaf et al. [6] (2020)
	CBC/PVA/zeolite	50	0.74	30wt%[a]	20wt%[a]	80	4.8	Altaf et al. [6] (2020)

KOH-doped	Qchitosan-PVA/LDH @CNT	1	2 (5 M KOH)	4	2	80	107	Gong et al. [7] (2019)
	Qchitosan-PVA/LDH	5	2 (6 M KOH)	4	2	60	97.8	Hu et al. [8] (2019)
	Qchitosan-PVA/3D-LDH	6	2 (5 M KOH)	4	2	60	73	Zhao et al. [9] (2021)
	PVA/HH/GA	5	3 (2 M KOH)	2	2	60	12.1	Gaur et al. [10] (2021)
	PVA-FP/[DimL]/GA	2.5	3 (5 M KOH)	0.5	0.5	70	48	Wang et al. [11] (2019)
	PVA/40.5% ZIF-8/GA	40.5	2 (6 M KOH)	2	1	60	191	This work

[a] Data shown in weight percentage. Catalyst loadings is not determine.
CBA- 4-carboxybenzaldehyde
AA-TiO_2- Amino acid functionalized titanium oxide
Qchitosan - Quaternized chitosan
LDH@CNT- Layered double hydroxide@ carbon nanotubes
3D-LDH- Three-dimensional hierarchial flower-like LDH
HH- Hair hydrolysate
FP/[DimL]- Pyridine functionalized geminal-imidazolium-type ionic liquid
*CBC- N-p-*carboxy benzyl chitosan
PVA-b-PVBTAC- Poly (vinyl alcohol-b-vinyl benzene trimethyl ammonium chloride)

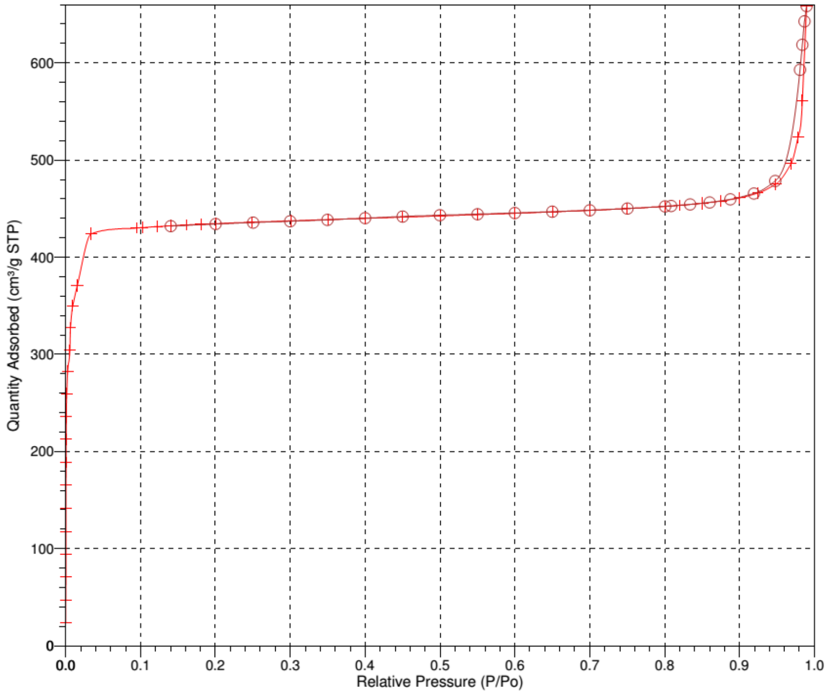

Figure S1. Nitrogen adsorption-desorption isotherm of ZIF-8 nanoparticles.

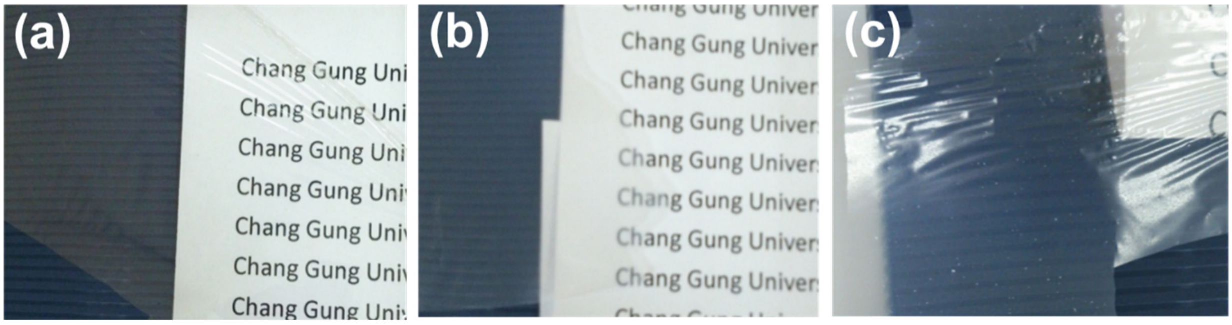

Figure S2. Photographic images of (a) PVA/GA, (b) PVA/40.5%ZIF-8/GA, and (c) PVA/45.4%ZIF-8/GA composite membranes.

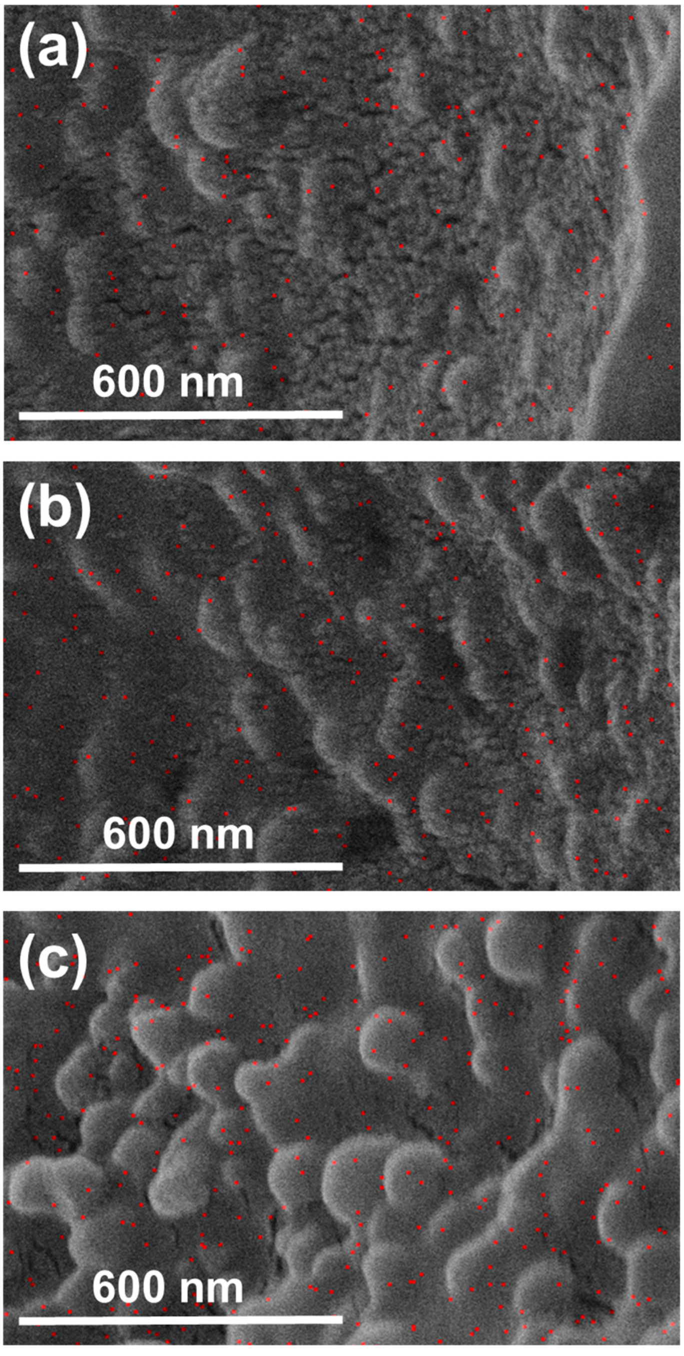

Figure S3. EDX mapping micrographs showing Zn distribution in (a) PVA/25.4%ZIF-8/GA, (b) PVA/40.5%ZIF-8/GA, and (c) PVA/45.4%ZIF-8/GA composite membranes.

Table S2. The weight percentages (determined from EDS) of PVA/ZIF-8/GA composites with various ZIF-8 loadings.

**ZIF loadings**	**0%**	**25%**	**40.5%**	**45.4%**
**Element**	**Wt (%)**	**Wt (%)**	**Wt (%)**	**Wt (%)**
C	72.47	76.96	68.46	68.21
O	27.53	14.48	18.04	16.27
Zn	-	8.56	13.50	15.52

References

[1] Y. Li, L. Liang, C. Liu, Y. Li, W. Xing, J. Sun, Self-Healing Proton-Exchange Membranes Composed of Nafion–Poly (vinyl alcohol) Complexes for Durable Direct Methanol Fuel Cells, Advanced Materials, 30 (2018) 1707146.

[2] D.u. Imaan, F.Q. Mir, B. Ahmad, Proton exchange membrane based on poly (vinyl alcohol) as support and alpha alumina as filler and its performance in direct methanol fuel cell, Journal of Environmental Chemical Engineering, 9 (2021) 106119.

[3] O. Gil-Castell, Ó. Santiago, B. Pascual-Jose, E. Navarro, T.J. Leo, A. Ribes-Greus, Performance of Sulfonated Poly(Vinyl Alcohol)/Graphene Oxide Polyelectrolytes for Direct Methanol Fuel Cells, Energy Technology, 8 (2020) 2000124.

[4] A.S. Rao, K.R. Rashmi, D.V. Manjunatha, A. Jayarama, V. Veena Devi Shastrimath, R. Pinto, Methanol crossover reduction and power enhancement of methanol fuel cells with polyvinyl alcohol coated Nafion membranes, Materials Today: Proceedings, 35 (2021) 344-351.

[5] S. Mohanapriya, G. Rambabu, S. Suganthi, S.D. Bhat, V. Vasanthkumar, V. Anbarasu, V. Raj, Bio-functionalized hybrid nanocomposite membranes for direct methanol fuel cells, RSC Advances, 6 (2016) 57709-57721.

[6] F. Altaf, R. Batool, R. Gill, M.A. Shabir, M. Drexler, F. Alamgir, G. Abbas, A. Sabir, K.I. Jacob, Novel N-p-carboxy benzyl chitosan/poly (vinyl alcohol/functionalized zeolite mixed matrix membranes for DMFC applications, Carbohydrate Polymers, 237 (2020) 116111.

[7] C. Gong, S. Zhao, W.-C. Tsen, F. Hu, F. Zhong, B. Zhang, H. Liu, G. Zheng, C. Qin, S. Wen, Hierarchical layered double hydroxide coated carbon nanotube modified quaternized chitosan/polyvinyl alcohol for alkaline direct methanol fuel cells, Journal of Power Sources, 441 (2019) 227176.

[8] Y. Hu, W.-C. Tsen, F.-S. Chuang, S.-C. Jang, B. Zhang, G. Zheng, S. Wen, H. Liu, C. Qin, C. Gong, Glycine betaine intercalated layered double hydroxide modified quaternized chitosan/polyvinyl alcohol composite membranes for alkaline direct methanol fuel cells, Carbohydrate Polymers, 213 (2019) 320-328.

[9] S. Zhao, W.-C. Tsen, C. Gong, 3D nanoflower-like layered double hydroxide modified quaternized chitosan/polyvinyl alcohol composite anion conductive membranes for fuel cells, Carbohydrate Polymers, 256 (2021) 117439.

[10] S.S. Gaur, P. Dhar, K.M. Wani, M. Srivastava, S. Sakurai, A. Kumar, V. Katiyar, Ion transfer channel network formed by flower and rod shape crystals of hair hydrolysate in poly(vinyl alcohol) matrix and its application as anion exchange membrane in fuel cells, Journal of Colloid and Interface Science, 587 (2021) 214-228.
[11] D. Wang, Y. Wang, J. Wang, L. Wang, Synthesized Geminal-imidazolium-type ionic liquids applying for PVA-FP/[DimL][OH] anion exchange membranes for fuel cells, Polymer, 170 (2019) 31-42.
